# Retraction Note: The acceptability of using IUDs among Egyptian nulliparous women: a cross-sectional study

**DOI:** 10.1186/s12905-025-04131-1

**Published:** 2025-11-14

**Authors:** Reham Refaat Elkhateeb, Eman Kishk, Ahmad Sanad, Haitham Bahaa, Abdel Rahman Hagazy, Kareem Shaheen, Enas Moustafa, Hahem Fares, Khalid Gomaa, Ahmad Mahran

**Affiliations:** 1 Obstetrics and Gynecology, Faculty of medicine Minia University, Maternity Hospital Minia University, Minia, Egypt; 2 Obstetrics and Gynecology, Faculty of Medicine Suez Canal University, Suez, Egypt; 3 Obstetrics and Gynecology Minia University, Minia, Egypt; 4 Obstetrics and Gynecology, Faculty of Medicine Minia University, Minia, Egypt; 5 Obstetrics and Gynecology at Maternity Hospital, Minia University, Minia, Egypt

**Retraction Note: BMC Women’s Health 20, 117 (2020)**

**https://doi.org/10.1186/s12905-020-00977-9**

This article has been retracted by the Editor. Following publication of this article, concerns were raised regarding the validity of data in Tables 1, 2, 3 and 4. The authors have been unable to provide an explanation for this or their raw data. In addition, they have been unable to provide a copy of their ethics approval documentation. The Editor therefore no longer has confidence in the results and conclusions of this article.

Author Reham Elkhateeb disagrees with this retraction. Authors Eman Kishk, Ahmad Sanad, Haitham Bahaa, Abdel Rahman Hagazy, Kareem Shaheen, Enas Moustafa, Hahem Fares, Khalid Gomaa and Ahmad Mahrandid not respond to correspondence from the Publisher about this retraction.

